# Practical Aspects of Medical Science

**Published:** 1894-04-28

**Authors:** 


### Practical Aspects of Medical Science.

### MENTAL AND PHYSICAL CONDITION OF SCHOOL CHILDREN.

From notes taken by a Select Committee investigating the mental and physical condition of school children, we learn that about 7 per cent. are "mentally dull," whilst 16 per cent. require special care and training. It appears that 50,000 children have been examined for this purpose by the committee above referred to, of which body Sir Douglas Galton is chairman. If sufficient funds are forthcoming the field of investigation is to be yet further extended and, it is hoped, that 100,000 cases will be examined with this object. It is only of late years that the question even of the very existence of the abnormal among school children has been raised at all, but more depends on definitely ascertaining facts regarding them than would appear at first sight. It is, indeed, of equal importance from a social, as from an educational, point of view. The Chairman of the Board of Investigation further enlarges on this question in the pages of a current monthly. "The rapid growth of our population is a distinguishing feature in the progress of England." He points out "this growth, partly, if not mainly, arises from the fact that with increased attention to hygienic conditions, the mortality of the population in the earlier stages of life has been diminished. This means that the proportion of sickly children reared is larger than was formerly the case, but it further means that a really appreciable number of feebly-gifted children are being brought up in every class of the social scale who would probably formerly have died in their early years."

These feebly-gifted and defective children are quite unfitted to compete either in school or in after life, urges our authority, with the average normal child; they are not even fitted for emigration, and whilst the healthy and the energetic go away to the colonies or to America, it is these dregs, so to speak, who are left on our hands, with all their tendencies to pauperism, starvation, vagrancy, and crime. Through them the State, of course, becomes heavily burdened—a burden which, as Sir Douglas Galton declares, expresses itself in the great masses of the unemployed. It was with the intention of definitely paving the way for further measures for really improving the average development, nuitrition, and potentiality for mental faculty that the investigation referred to at the commencement of this paper was first brought about; and through this means eventually doing something to lessen pauperism and social failure by removing causes which lead to social and moral degeneration among the populace. To obtain information as to the exact condition of the children of a locality is for these reasons as important a matter for the statistics which govern hygiene as the knowledge of the death-rate of a given locality, because, as our authority says, the facts relating to their maldevelopment appear to show an accompaniment of mental dulness. The penalty of neglect, foul air, and low nutrition is shown during the child's existence, if not by its termination.

The actual method which was employed to determine the normal from the abnormally minded among the school children who were thus examined required no inconsiderable insight and powers of observation on the part of the examiners. Dr. Francis Warner's mode of procedure is described as follows: The pupils were first observed as they stood in rank, usually in small sections at a time. The inspector, standing in front of each child in turn, held a shilling for him to look at, so as to fix his eyes, and thus obtain a full face as well as a profile view of each side, noting the features separately, the cranium, the expression, and the muscular action of the parts of the face, the eye movements, and other points. *

We are told that the trained observer quite readily can read these signs as presented by the scholars, and determine with a quick certainty the thin boundary line which exists between capacity and incapacity in the mental balance. Dr. Francis Warner has devoted himself no less than eighteen years to studying the varied and various indications of mental status in children. After the examination of the children collectively, further and more exhaustive tests were individually applied. A paper was then drawn up on which each is correspondingly incribed in schedule form. In this way, Sir Douglas Galton tells us, is the tabular presentation of the cases attained, from which actuarial analyses and groupings can most accurately be prepared.

The accompanying table, extracted from the Report on the Physical and Mental Condition of English and Irish Children, shows us the percentage of abnormal children upon the number of children seen in certain classes of schools in England in 1890-91:—

| School, &c. | Number seen. | | | Percentage of Cases Noted as Abnormal. | | | | | |
|---|---|---|---|---|---|---|---|---|---|
| | Boys. | Girls. | Total. | English. | | Irish. | | Jewish. | |
| | | | | B. | G. | B. | G. | B. | G. |
| Poor Law Schools | 5,884 | 3,947 | 9,831 | 21·8 | 17·3 | 27·8 | 19·1 | | |
| Certified Industrial Schools | 1,588 | 407 | 1,995 | 21·1 | 20·4 | 49·7 | 28·2 | | |
| Homes and Orphanages | 774 | 1,049 | 1,823 | 22·2 | 17·7 | | | | |
| Public Elementary and Day Schools | 18,137 | 16,854 | 34,991 | 19·2 | 15·0 | 24·0 | 16·4 | 17·8 | 13·9 |
| Other schools | 501 | 886 | 1,387 | | | | | | |
| Total | 26,884 | 23,143 | 50,027 | | | | | | |

From this we see that the percentage of deviations from the normal in children averages somewhat higher than was at first stated. We are glad to learn that foremost in the vanguard of those centres which already have agreed to make special arrangements to meet the requirements of these specialised cases are the School Boards of London, Leicester, and Birmingham, but it is only to be surmised that others will readily follow in their wake when the work of this committee under Sir Douglas Galton's able chairmanship becomes more definitely recognised.

It is beyond question or doubt that when boards are brought face to face with the facts concerning the mentally deficient among their respective children, more detailed attention should, and will be given to the mental and physical condition of such cases.

It is interesting to learn that among all the children who were brought under the examination, Dr. Warner found the greater mental development, as also the more perfect physical formation among the children of Semitic races in Whitechapel than in any other part of Great Britain.

* See Dr. Warner's "Lectures on Mental Faculty."

